# A Dipole with Reflector-Backed Active Metasurface for Linear-to-Circular Polarization Reconfigurability

**DOI:** 10.3390/ma15093026

**Published:** 2022-04-21

**Authors:** Ruan van Aardt, Johan Joubert, Johann W. Odendaal

**Affiliations:** Department of Electrical, Electronic and Computer Engineering, University of Pretoria, Pretoria 0002, South Africa; ru.vanaardt@gmail.com (R.v.A.); wimpie.odendaal@up.ac.za (J.W.O.)

**Keywords:** metasurface, reflector-backed, polarization diversity, reconfigurable polarization, dipole antenna

## Abstract

In recent years, significant advances have been made in diversifying the capabilities of communication systems by using reconfigurable antennas. There are many types of reconfigurable antennas—to achieve pattern, frequency, or polarization reconfigurability. These antennas are reconfigured either by the mechanical rotation of surfaces or by enabling or disabling specific sections of the structure using electrical switches. This paper focuses on the concept of a polarization reconfigurable antenna based on an active reflector-backed metasurface. An antenna system based on an active reflector-backed metasurface combined with a planar dipole is designed to achieve reconfigurable polarization. The polarization of the designed antenna can be switched between linear and circular polarization states using positive-intrinsic-negative diodes located in the unit cell elements of the metasurface. The measured results correlate well with the simulated results. The antenna has a physical size of 308 × 162 × 35 mm^3^ with an impedance bandwidth of 4.5% in the linear state and 7% in the circular state, as well as an axial ratio bandwidth larger than 8.3%.

## 1. Introduction

The communication industry has rapidly evolved over the last decade because of a focus on a more connected world, with an emphasis on wireless technologies. A major challenge in the industry has been the demand for networks to have more capacity and throughput while operating with spectral efficiency. Reconfigurable antennas have been investigated to address this challenge because they can provide the required diversity [1].

As an active research field, reconfigurable antennas still allow for significant innovations, which can have a major impact on the communication industry. Even though reconfigurable antennas have their challenges, they still provide great advantages in the communication domain compared to traditional antennas. Reconfigurable antennas have shown significant improvements in communication systems, specifically in MIMO based systems [2]. By using certain reconfigurable antennas, it is possible to improve the data throughput, the signal to noise ratio (SNR), and the bit error rate. Not only does a reconfigurable antenna improve performance characteristics, but it also makes the antenna multifunctional [3].

Some reconfigurable antenna designs achieve reconfigurability by modifying the structure of the radiating element. This is done by introducing switching elements on the radiating element, which enables the user to change the properties of the antenna through a controller. However, by modifying the antenna structure with the addition of switching elements, the antenna performance will be degraded. As shown in [2,3,4,5], this degradation can be rectified through the addition of inductive chokes to still achieve the desired antenna performance. In [4], a microstrip patch is connected to 12 stubs through varactor diodes. By altering the bias voltages of the diodes, specific stubs can be activated to enable circular and linear polarization configuration states. An impedance bandwidth of 40% is achieved with a narrow axial ratio bandwidth over multiple frequencies. A similar approach is taken in [5], where a reflector-backed wheel-shaped monopole antenna is stub-loaded. Again, by varying the bias voltages, circular and linear polarization states can be realized, achieving impedance bandwidths of 5.4% and 28.6%, respectively, in the linear and circular configuration states. An axial ratio bandwidth of 15.4% is achieved in the circular configuration.

Reconfigurability can also be accomplished by modifying the feed network by introducing switchable segments that can alter the phase and magnitude of the input signal [6,7,8,9]. These designs produce antennas with wide bandwidths and high gains. With most of these methods, reconfigurable polarization is the focus of the designs. In [6], a dielectric resonator antenna (DRA) is made reconfigurable through the addition of a feeding cross slot network with embedded positive-intrinsic-negative (PIN) diodes. The antenna is capable of right-hand circular polarization (RHCP), left hand circular polarization (LHCP), and linear polarization (LP) states. The antenna has impedance bandwidths of 30% and 25%, respectively, in the linear and circular polarization states, while an axial ratio bandwidth of 21% is achieved.

Metasurfaces are two-dimensional arrays composed of subwavelength structures that can be designed to have specific responses on electromagnetic waves. At microwave frequencies, planar metallic arrays of unit elements etched on low-loss dielectric substrates are typically used as metasurfaces to modify the amplitude, phase, and polarization of electromagnetic waves. These single-layer monoatomic designs are relatively easy to design and manufacture. Recent research shows that anisotropic plasmonic, diatomic, and polyatomic metasurfaces will allow even more degrees of freedom than planar monoatomic metasurfaces and will make it possible to realize improved electromagnetic functionalities [10,11,12].

Single-layer monoatomic metasurfaces have been used to alter the polarization of an incident wave [13,14,15,16,17], either in a transmission or reflective configuration. In [13], a multilayered metasurface with truncated square patch unit cells is used to achieve polarization reconfigurability. By mechanical rotation of the surface RHCP, LHCP, and LP states can be achieved. In [14], this metasurface is implemented with a slot radiating element to create a reconfigurable antenna. To alter the polarization states, the surface is rotated as originally described in [13]. The antenna achieves an impedance bandwidth of 25% and 37%, respectively, in the linear and circular states. An axial ratio bandwidth of 14% is achieved. At microwave frequencies, these metasurfaces can be made reconfigurable through the addition of switchable elements. The metasurface can be used to add the element of reconfigurability within an antenna system [15,18,19,20,21,22]. Micro-electromechanical system (MEMS) devices can also be used to reconfigure metasurfaces. In [18], a metasurface with a 4 × 4 square unit cell array was used in conjunction with a square patch antenna. The unit cells are connected to each other through MEMS switches where they can be controlled independently to achieve pattern, frequency, and polarization diversity. The antenna achieves an impedance bandwidth of 1.8% for both linear and circular configurations and an axial ratio bandwidth of 2.4%. The authors also show that an array of radiators can be implemented with such a reconfigurable surface. Another antenna with polarization diversity based on a reconfigurable metasurface is discussed in [19]. The metasurface is designed with a double layered elliptical unit cell structure and is capable of switching the polarization state from linear to circular. This surface is used in front of the aperture of a horn antenna. The reconfigurability is achieved through PIN diodes: in the off state, the antenna is configured in a circular polarization state and a linear polarization state in the on state. An impedance bandwidth of 21% and 23% is achieved in the linear and circular states with an axial ratio bandwidth of 14%. In all the previously mentioned examples, a unidirectional antenna radiating element was used, with the reconfigurable metasurface as a superstrate to control the polarization. An alternative method of utilizing a metasurface for reconfigurability is in a reflective configuration for omnidirectional radiating elements where the metasurface is backed with an electric reflective surface. In [15], an active reflective metasurface is presented that could convert a linearly polarized incident wave to a circularly polarized reflected wave. The reflected wave can be switched from LHCP to RHCP, at distinct frequencies. In [20], a double layered metasurface consisting of rectangular patch unit cells is utilized together with a reflector and a CPW (co-planar waveguide) fed monopole. The antenna can switch between an RHCP state and an LHCP state over multiple narrow frequency bands using varactor diodes. The antenna achieves an impedance bandwidth of 12.8% and an axial ratio bandwidth of 2.85%. This is one of very few reported studies on a reflector-backed active metasurface antenna. To the authors’ knowledge, no previous results have been published to show that linear to circular polarization reconfigurability can be achieved with a reflector-backed active metasurface. Antennas using a reflector-backed active metasurface to achieve reconfigurability have potential advantages over that of antennas with transmission metasurfaces. The biasing circuitry may be easier to implement behind the reflector, which will minimize interference on the antenna. Scalability might also be easier for arrays of radiators. The main contribution of this paper is to illustrate that a reflector-backed active metasurface can also be used for linear-to-circular polarization reconfigurability. A printed dipole radiator with a reflector-backed active metasurface was designed and is discussed. Measured results are presented to confirm the validity of the design.

## 2. Design Procedure

**Figure 1:**
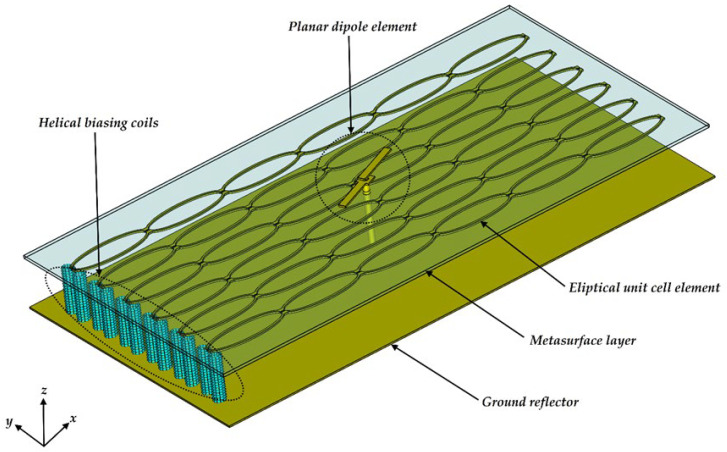
Three-dimensional antenna structure.

A design was performed for a center frequency of 2.4 GHz. The three-dimensional antenna structure is shown in Figure 1. The antenna consists of three components, namely the printed dipole radiating element, the active metasurface, and the electric reflector. The dipole element is fed through the reflector and the metasurface with a coaxial cable. The metasurface was designed with a 6 × 6 elliptical unit cell array. The PIN diode switches are not drawn in Figure 1, but the unit cells were specifically designed to operate with a 7 × 6 array of PIN diodes soldered across the small gaps in the elliptical unit cell profiles at the edges of each unit cell. The antenna has reconfigurable polarization with two polarization states: LP and RHCP. The reconfigurability is achieved through the PIN diodes on the unit cell edges, which either short the two elliptical halves of the unit cell or create an open circuit between the elliptical halves. The PIN diodes are biased appropriately to operate in a forward biased configuration. In the off position a circular polarization state is achieved, and in the on position a linear state is achieved. The diodes are connected in parallel and are biased at the edge of the metasurface through helical coils, which act as RF chokes. The PIN diodes used for the antenna system are BAR65 diodes with a junction voltage and current draw of 0.93 V and 100 mA respectively. The metasurface and the planar dipole antenna were both manufactured on ROGERS 4003C substrate with a height of 0.813 mm. The entire size of the final antenna is 308 × 162 × 35 mm^3^_._

**Figure 2:**
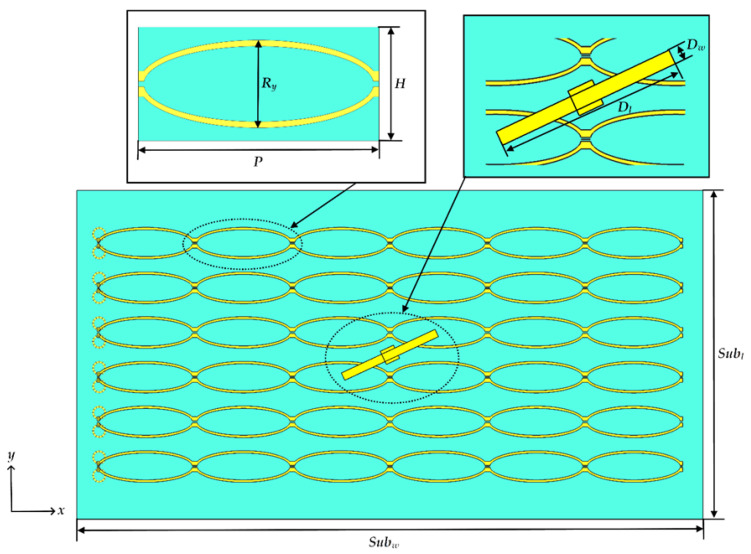
Top view of the antenna.

**Figure 3:**
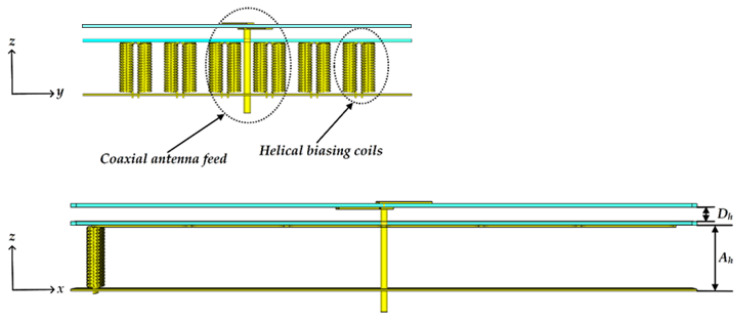
Side views of the antenna.

A top view of the antenna is shown in Figure 2, with a focus on the unit cell geometry and the dipole element. Figure 3 presents side views of the antenna to clearly show the coaxial feed and helical biasing coils.

The reconfigurable reflector-backed metasurface in this paper combines the linearly polarized field radiated directly from the dipole (***E_i_***) in the main beam direction with the reflected fields (***E_r_***) from the reflector-backed metasurface to realize a total vector field (***E_T_*** = ***E_i_*** *+ **E_r_***) that is either linearly polarized or circularly polarized over the same frequency range. To achieve a circularly polarized radiated wave, the total electric field components, *E_Tx_* and *E_Ty_*, should have equal magnitude with a phase difference of 90° between the two components.

An initial single unit cell for the reflector-backed metasurface was designed assuming an infinite surface in the *xy*-plane. The metasurface geometry is similar to the structure used in [19] with a meandering elliptical unit cell. The design was accomplished using full wave electromagnetic simulations in CST Studio Suite 2020 (Dassault Systèmes Simulia, Johnston, R, USA) using perfect electric and magnetic boundary conditions and a floquet port. The Z_max_ port was positioned in front of the unit cell and excited the unit cell through floquet modes and monitored the reflected fields in front of the unit cell. To design the unit cell the critical parameters were determined through a parametric study. The incident polarization angle remained 45° throughout the initial design of the reflector-backed metasurface unit cell. The method of operation is explained through the effect of the metasurface on the reflected field components. The impedance seen by the two reflected linear field components differs from each other and is affected by the state of the switches at the edges of the unit cells. When the sides of the unit cells are open, the surface acts as if there is a shunt capacitance applied to the *E_ry_* component. This shunt capacitance creates a 90° phase shift for the *E_ry_* component and in effect creates a circularly polarized reflected wave. When the sides of the unit cell are closed, it eliminates the shunt capacitance that was applied to the *E_ry_* component and then the surface reflects an x-directed linearly polarized wave. The critical parameters for adjusting the characteristics of the unit cell include the unit cell width, the unit cell height, the spacing between the reflector and the metasurface, and the radii of the elliptical microstrip line. The axial ratio was calculated for the reflected field using equation (1), where *δ* is the phase difference between the *E_rx_* and *E_ry_* reflected field components [15].

(1) $$AR\left(dB\right)=20log_{10}\frac{\sqrt{\left(E_{rx}{}^{2}+E_{ry}{}^{2}+\sqrt{E_{rx}{}^{4}+E_{ry}{}^{4}+2E_{rx}{}^{2}E_{ry}{}^{2}cos2\delta }\right)}}{\sqrt{\left(E_{rx}{}^{2}+E_{ry}{}^{2}-\sqrt{E_{rx}{}^{4}+E_{ry}{}^{4}+2E_{rx}{}^{2}E_{ry}{}^{2}cos2\delta }\right)}}$$

**Figure 4:**
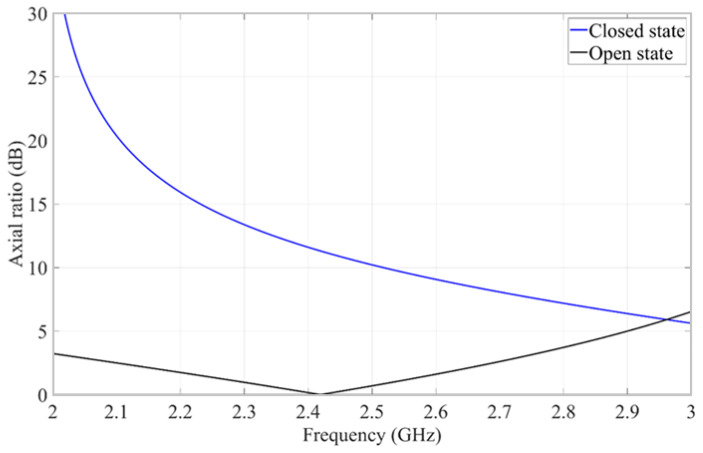
Simulated axial ratio of the reflected fields from the designed reflector-backed metasurface in a closed and open state.

Using the results of the parametric study, a parameter optimization was done to design the reflector-backed metasurface. In the open state, the reflector-backed metasurface operates in a circular configuration, and the reflected *E_rx_*- and *E_ry_*-field components have equal magnitude and 90° phase difference. In the closed state, the reflected wave consists of only an *E_x_*-field component. The simulated axial ratio of the reflected fields from the designed reflector-backed metasurface in an open state and closed state is shown in Figure 4. The reflector-backed metasurface in an open state produces a 3 dB axial ratio bandwidth of 30%, from 2.03 GHz to 2.75 GHz. The closed state produces a high axial ratio, which shows that the reflector-backed metasurface will be close to linearly polarized within the operating band for this state.

The planar dipole element was also initially designed separately (without the reflector-backed metasurface) using CST Studio Suite 2020 (Dassault Systèmes Simulia, Johnston, R, USA). The planar dipole length and width were determined to achieve minimum reflection at the design frequency of 2.4 GHz, for a 50 Ω coaxial feed line.

The final design with the printed dipole integrated with the reflector-backed metasurface was then also performed in CST Studio Suite 2020 (Dassault Systèmes Simulia, Johnston, R, USA). This final step in the design procedure included sequential parameter tuning to determine a set of final dimensional parameters (of the printed dipole and the reflector-backed metasurface) that provided acceptable axial ratio, impedance bandwidth, and radiation patterns. The dimensions of the initially designed printed dipole and reflector-backed metasurface were used as starting values for the optimization process. The rotation angle of the printed dipole and the air gap between the metasurface and the reflector are two of the crucial parameters to achieve total radiated fields that are respectively circularly and linearly polarized in the open and closed states of the PIN diodes. The total radiated fields are a combination of the direct radiated fields from the printed dipole, ***E_i_***, and the reflected fields from the reflector-backed metasurface, ***E_r_***. The design parameters of the antenna and their final values are tabulated in Table 1.

## 3. Results and Discussion

**Figure 5:**
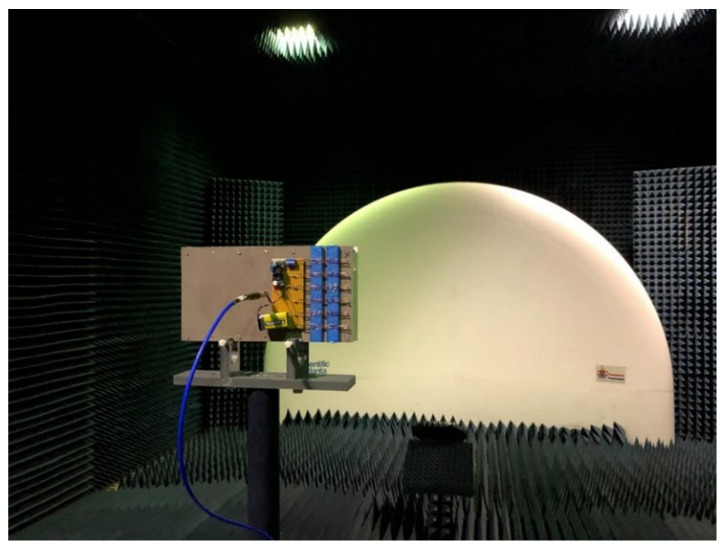
Antenna measurement set-up.

The antenna was assembled and measured at the compact antenna range at the University of Pretoria. A photograph of the antenna mounted in the compact antenna range is shown in Figure 5.

**Figure 6:**
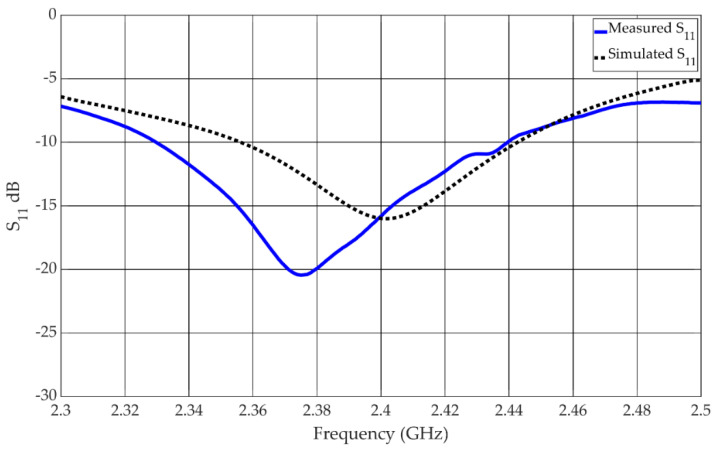
Measured and simulated reflection coefficient of the antenna in the linear state.

**Figure 7:**
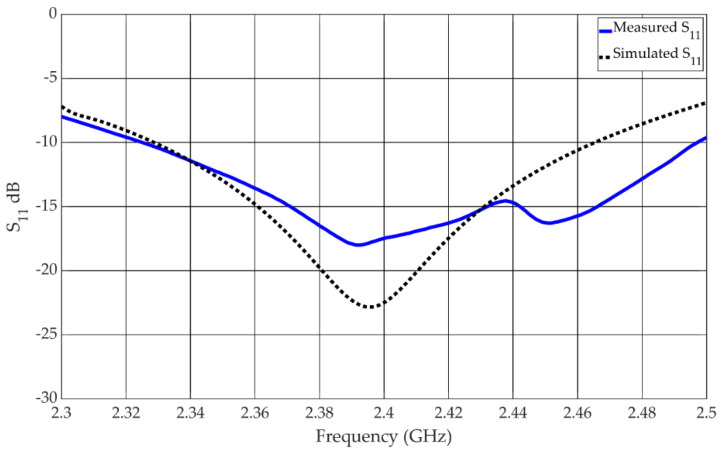
Measured and simulated reflection coefficient of the antenna in the circular state.

The reflection coefficient of the antenna in the linear state is shown in Figure 6. The −10 dB impedance bandwidth was measured to be from 2.33 GHz to 2.44 GHz, which equates to a bandwidth of 4.5%. The reflection coefficient for the antenna in the circular state is shown in Figure 7. The measured −10 dB impedance bandwidth is 7%, spanning the frequency range from 2.328 GHz to 2.498 GHz.

**Figure 8:**
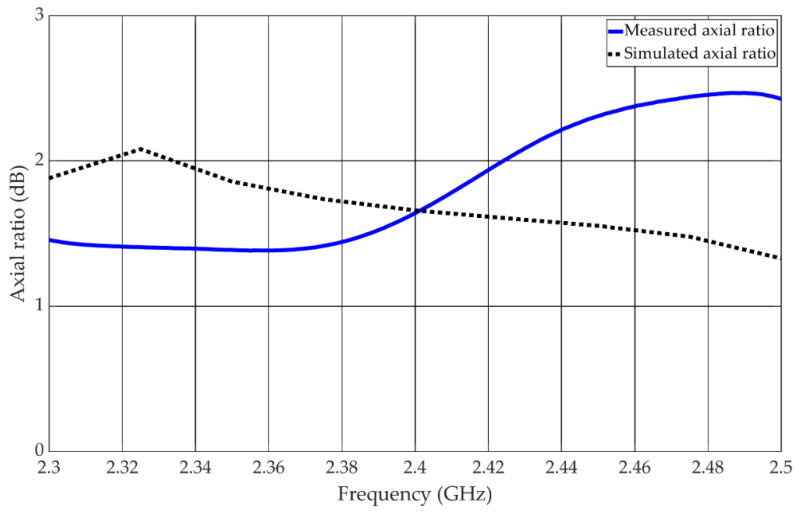
Axial ratio of the antenna in circular state.

The measured axial ratio of the antenna is shown in Figure 8. The axial ratio bandwidth is lower than 3 dB throughout the measurement range and is thus larger than 8.3%.

**Figure 9:**
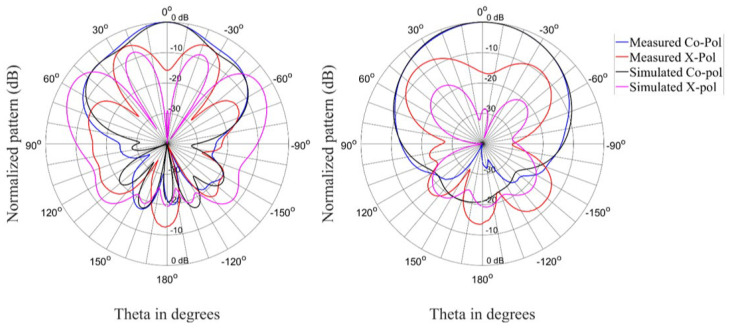
Radiation patterns (Phi = 0° and Phi = 90°) of the antenna in the linear state at 2.35 GHz.

**Figure 10:**
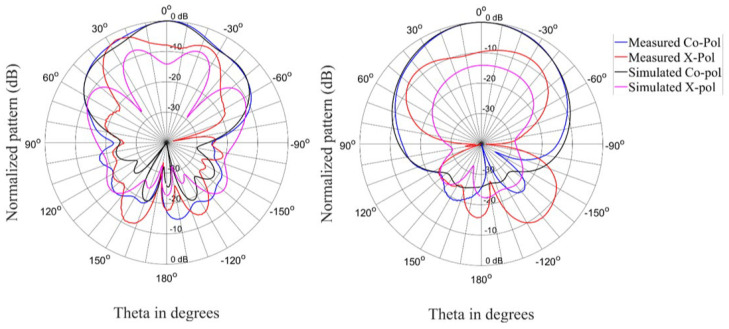
Radiation patterns (Phi = 0° and Phi = 90°) of the antenna in the linear state at 2.4 GHz.

**Figure 11:**
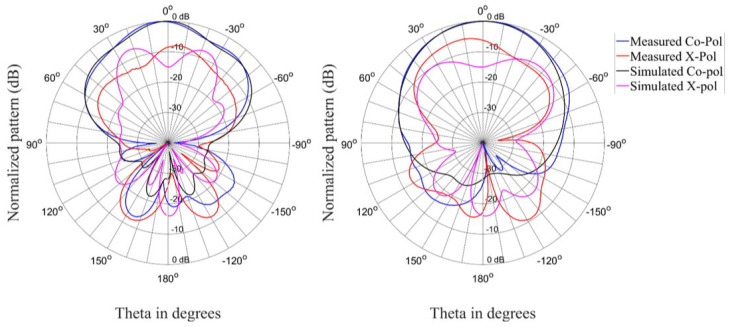
Radiation patterns (Phi = 0° and Phi = 90°) of the antenna in the linear state at 2.45 GHz.

The radiation patterns for the antenna in the linear state are shown in Figure 9, Figure 10 and Figure 11 for three different frequencies at the lower edge, center, and upper edge of the operating frequency band. There is acceptable correlation between the simulated and measured co-polarized radiation patterns. The correlation between the simulated and measured cross-polarized patterns is similar in terms of the general shape of the radiation patterns, but the measured levels are higher.

**Figure 12:**
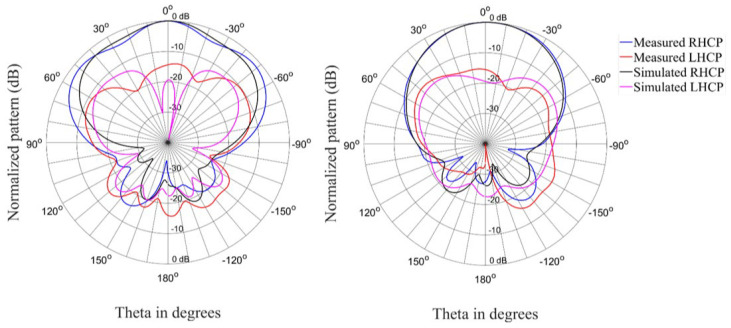
Radiation patterns (Phi = 0° and Phi = 90°) of the antenna in the circular state at 2.35 GHz.

**Figure 13:**
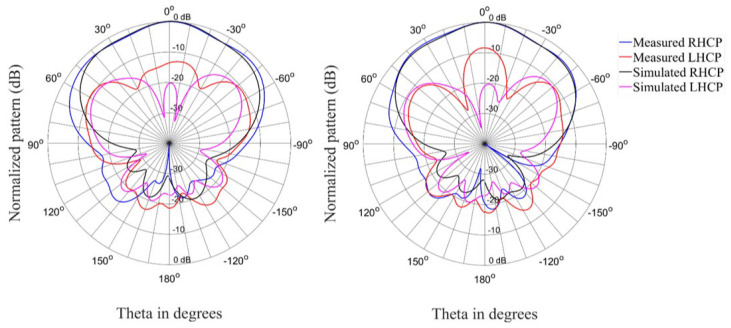
Radiation patterns (Phi = 0° and Phi = 90°) of the antenna in the circular state at 2.4 GHz.

**Figure 14:**
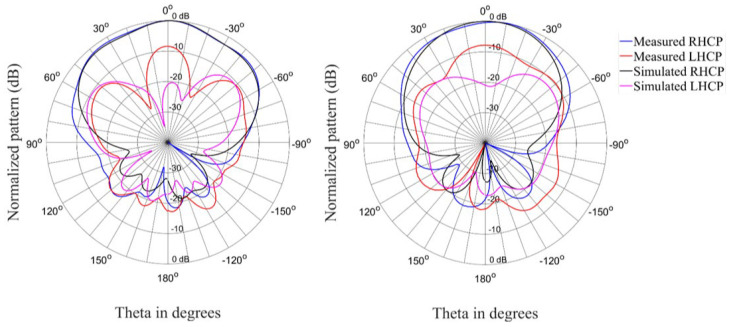
Radiation patterns (Phi = 0° and Phi = 90°) of the antenna in the circular state at 2.45 GHz.

Figure 12, Figure 13 and Figure 14 show the radiation patterns for the antenna in the circular state. The correlation between simulated and measured results is acceptable for the co-polarized (RHCP) and cross-polarized (LHCP) patterns.

The overall performance of the antenna is satisfactory, but there are some discrepancies between the measured and simulated results. These discrepancies in the performance can partly be attributed to variations in the coils due to the imperfect manufacturing methods used. The coils were required to bias the diodes as normal wiring altered the performance of the antenna. Coils were introduced to normalize the performance—this produced satisfactory results in simulation but not in measurement. There may also be more complex interactions via resonant modes that are created from the reflections of the coils within the cavity between the ground plane and the metasurface. This can be observed in the reflection coefficients shown in Figure 6 and Figure 7 of both linear and circular states of the antenna where resonant effects can be observed around 2.44 GHz. Although somewhat degraded, the measured data of the antenna did satisfactorily demonstrate the concept of creating a reflector-backed active metasurface to create an antenna with polarization reconfigurability with electrical switches.

## 4. Conclusions

Results of a working antenna prototype are presented with reconfigurable polarization which is accomplished through a reflector-backed active metasurface, which is combined with a planar dipole radiating element. The antenna can achieve linear horizontal and right-hand circular polarization states by activating switchable elements on the antenna. Reconfigurability is achieved with PIN diodes on the unit cell elements of the reflector-backed metasurface.

The antenna has a physical size of 308 × 162 × 35 mm^3^ and an impedance bandwidth of 4.5% in the linear state, 7% in the circular state, and an axial ratio bandwidth larger than 8.3%. This gives the antenna an effective bandwidth of 4.5% in the linear state and 7% in the circular state. The simulated results of the antenna showed unidirectional patterns with reasonable cross-polar performance in both linear and circular states. However, the measured cross-polar results were unfortunately degraded by the imperfect implementation of the helical coils.

A comparison of the various types of antennas with reconfigurable polarization is presented in Table 2. Most of the antennas are reconfigurable in multiple polarization states where most of the antennas are implemented with an active transmittive metasurface [14,18,19]. Only one author used a similar reflector-backed method to design a reconfigurable antenna [20]. The antenna was reconfigurable for two circular polarization states, while the antenna proposed in this paper can achieve both linear and circular polarization states. The proposed antenna also achieves a better effective bandwidth than the antenna in [20].

Consequently, the proposed antenna is one of a few antennas that are based on a reflector-backed active metasurface and, to the knowledge of the authors, the only one capable of both circular and linear polarization states. The antenna achieved performance comparable with other antennas with reconfigurable polarization.

## Figures and Tables

**Table 1:** Antenna parameter set.

Parameter	Description	Value
*A_h_*	Air gap between unit cell and reflector	27 mm
*P*	Unit cell width	48 mm
*H*	Unit cell height	22 mm
*R_y_*	Unit cell ellipse *y* radius	7.8 mm
*R_x_*	Unit cell ellipse *x* radius (*R_x_* = *P*/2)	24 mm
*C_h_*	Connection block height	2 mm
*C_w_*	Connection block width	2 mm
*S_h_*	Switch height	1 mm
*S_w_*	Switch width	1.2 mm
*U_w_*	Unit cell microstrip width	1.2 mm
*Sub_h_*	Substrate height	0.813 mm
*Sub_w_*	Substrate width	308 mm
*Sub_l_*	Substrate length	162 mm
*Rot_d_*	Rotational angle of dipole	25°
*D_h_*	Air gap between surface and dipole	6 mm
*D_w_*	Dipole width	4 mm
*D_l_*	Dipole length	50.85 mm
*G_x_*	Extra substrate length	10 mm

**Table 2:** Comparison of antennas with reconfigurable polarization.

Ref.	Reconfiguration Method	Polarization States	Impedance BW (%)		Cross-Polar Discrimination (dB)		AR BW (%)	Effective BW (%)
			Lin.	Circ.	Lin.	Circ.		
[5]	Alter radiating element structure	LHCP, RHCP, LP	2.6,5.4	28.6	>3	>18	15.4	15.4
[6]	Alter feed network structure	LHCP, RHCP, LP	30	25	>20	>20	21	20
[14]	Rotate metasurface structure	LHCP, RHCP, LP	25	37	50	15	14	11
[18]	Alter metasurface elements	CP, LP	1.8	1.8	Not given	Not given	2.4	1.6
[19]	Alter metasurface elements	LHCP, LP	21	23	10	>18	14	13
[21]	Alter metasurface elements	LHCP, RHCP	N/A	17	N/A	>15	4.58	1.6
[20]	Alter reflector-backed metasurface elements	LHCP, RHCP	N/A	12.8	N/A	>20	2.85	2.85
This work	Alter reflector-backed metasurface elements	RHCP, LP	4.5	7	7	11	8.3	4.5

## Data Availability

The data presented in this study are on request from the main author.
